# Author Correction: Prefrontal cortical ChAT-VIP interneurons provide local excitation by cholinergic synaptic transmission and control attention

**DOI:** 10.1038/s41467-020-14315-y

**Published:** 2020-02-04

**Authors:** Joshua Obermayer, Antonio Luchicchi, Tim S. Heistek, Sybren F. de Kloet, Huub Terra, Bastiaan Bruinsma, Ouissame Mnie-Filali, Christian Kortleven, Anna A. Galakhova, Ayoub J. Khalil, Tim Kroon, Allert J. Jonker, Roel de Haan, Wilma D. J. van de Berg, Natalia A. Goriounova, Christiaan P. J. de Kock, Tommy Pattij, Huibert D. Mansvelder

**Affiliations:** 1 Department of Integrative Neurophysiology, Center for Neurogenomics and Cognitive Research (CNCR), Vrije Universiteit, Amsterdam Neuroscience, The Netherlands; 2 Department of Anatomy and Neurosciences, Amsterdam UMC, Vrije Universiteit, Amsterdam Neuroscience, The Netherlands; 3 Present Address: Department of Anatomy and Neurosciences, Clinical Neuroscience, Amsterdam UMC, Vrije Universiteit, Amsterdam Neuroscience, The Netherlands; 4 Present Address: MRC Centre-Developmental Neurobiology, King’s College London, London, UK

**Keywords:** Neuroscience, Neural circuits, Synaptic transmission

Correction to: *Nature Communications* 10.1038/s41467-019-13244-9, published online 21 November 2019.

The original version of this Article contained an error in the spelling of the author Wilma D.J. van de Berg, which was incorrectly given as Wilma D.J. van den Berg. This has now been corrected in both the PDF and HTML versions of the Article.

