# Supplementary material for: Prefrontal cortical ChAT-VIP interneurons provide local excitation by cholinergic synaptic transmission and control attention
Source: Nat Commun. 2019 Nov 21;10:5280. doi: 10.1038/s41467-019-13244-9 (PMC6872593; doi:10.1038/s41467-019-13244-9)
Supplement: Supplementary file 1 — Supplementary Information [file 41467_2019_13244_MOESM1_ESM.pdf]

Supplementary figures

Prefrontal cortical ChAT-VIP interneurons provide local excitation by cholinergic synaptic transmission and control attention

by
Joshua Obermayer[1]†, Antonio Luchicchi[1,5] †, Tim S. Heistek[1], Sybren F. de Kloet[1], Huub Terra[1], Bastiaan Bruinsma[1], Oissame Mnie-Filali[1], Christian Kortleven[1], Anna A. Galakhova[1], Ayoub J.Khalil[1], Tim Kroon[1,6], Allert J. Jonker[2], Roel de Haan[1], Wilma D.J. van den Berg[2], Natalia A. Goriounova[1], Christiaan P.J. de Kock[1], Tommy Pattij[2*], Huibert D. Mansvelder[1*].

[1]Department of Integrative Neurophysiology, Center for Neurogenomics and Cognitive Research (CNCR), Vrije Universiteit, Amsterdam Neuroscience, The Netherlands.
[2]Department of Anatomy and Neurosciences, Amsterdam UMC, Vrije Universiteit, Amsterdam Neuroscience, The Netherlands.

Supplementary Figure 1: Layer 1 interneurons receive cholinergic miniature Excitatory PostSynaptic Currents (mEPSCs).

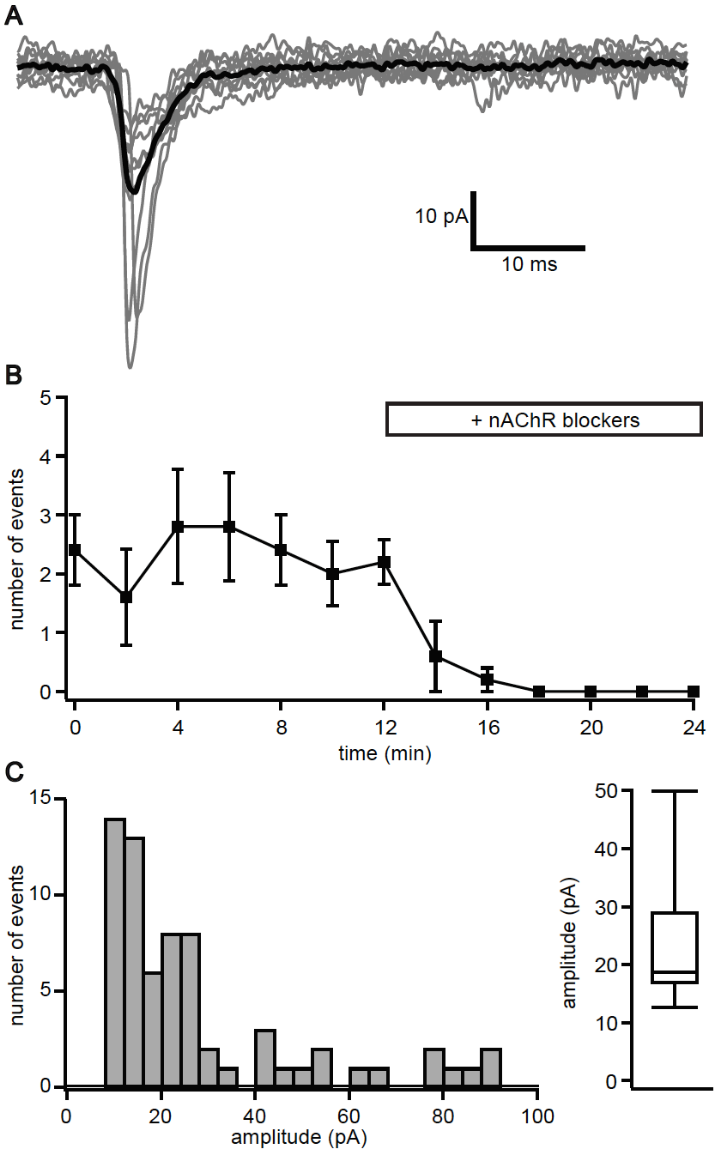

(A) Example traces of mEPSCs (grey) and average current trace (black). Miniature EPSCs were recorded in mPFC layer 1 interneurons in acute brain slices of adult wildtype Bl6 mice in the presence of blockers of voltage-gated sodium channels, AMPA-Rs, NMDA-Rs and GABA-Rs. (bath solution contained 1 μM TTX, 10 μM DNQX, 25 μM AP5, 10 μM gabazine, 4 mM calcium. The intracellular solution contained CsCl).

(B) The number of mEPSCs observed in two minute time bins is rapidly reduced to zero when antagonists (MLA and DHβE) of nicotinic Acetylcholine receptors (nAChRs) are applied (n=5 of 5 recorded neurons). Summary plots show averages ± SEM.

(C) left: Amplitude distribution of all recorded mEPSCs (n=5 neurons). Despite the modest number of mEPSCs (n=67 events) recorded in total, the histogram might suggest multiple amplitude peaks (quanta). Right: average mEPSC amplitude. Box plot centre line: median; bounds of box: 25th to 75th percentile; whiskers: min-max data bound.

Supplementary Figure 2: Morphology of dendrites and axons of ChAT-VIP neurons.

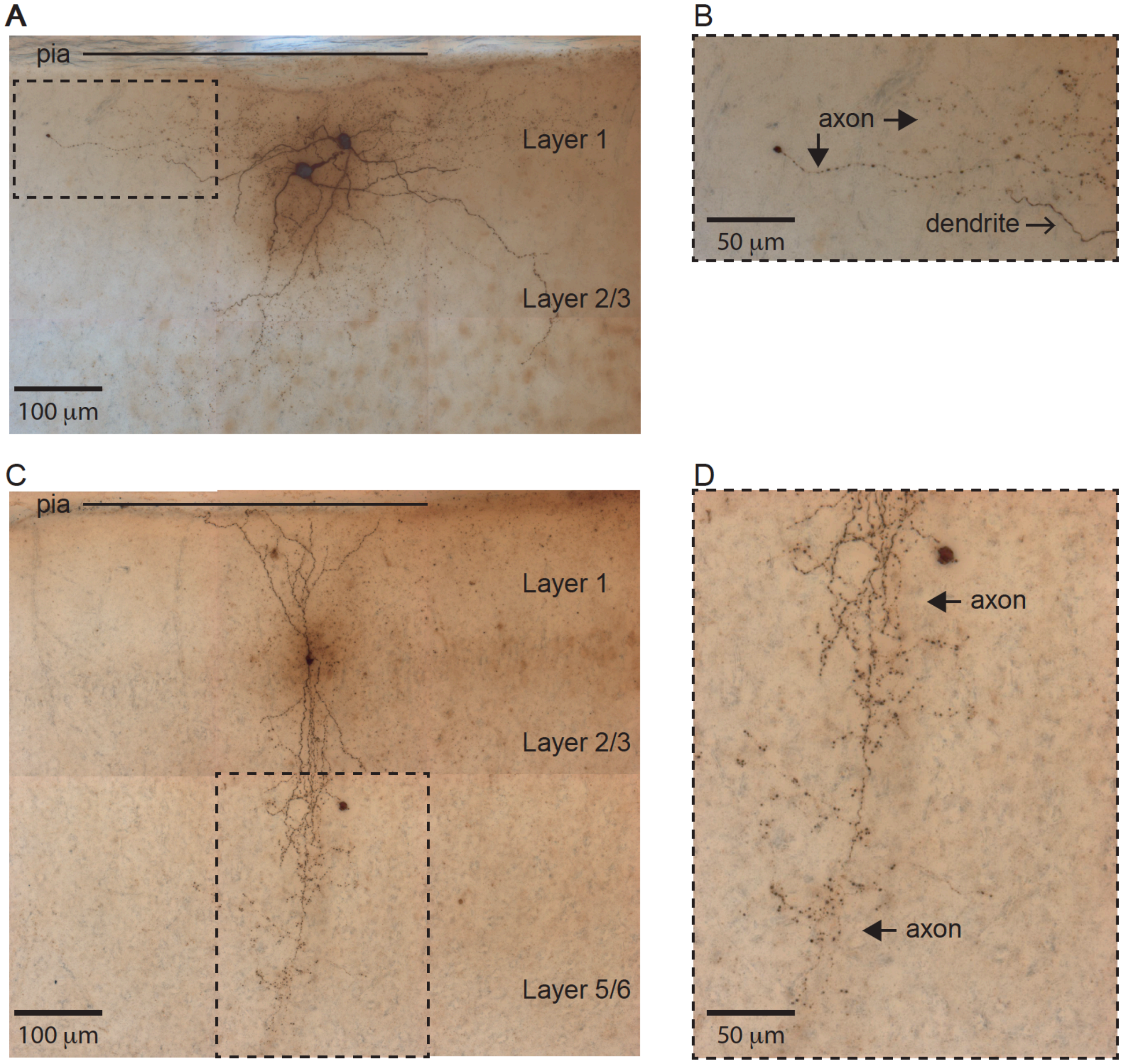

(A) Biocytin-filled ChAT-VIP neuron located on the border between Layer 1 and Layer 2 in a mouse mPFC brain slice showing multi-polar soma/dendritic morphology. Axons spread throughout layer 1.

(B) Magnification of the rectangular boxed region in (A) showing parts of the axon and dendrite.

(C) Biocytin-filled ChAT-VIP neuron in a mouse mPFC brain slice showing bipolar soma/dendritic morphology and axonal projections to deep layers 5 and 6.

(D) Magnification of the rectangular boxed region in (C) showing the axonal trajectory in layers 5 and 6.

Supplementary Figure 3: ChAT-VIP interneurons express ChAT, GAD, VIP and CR.

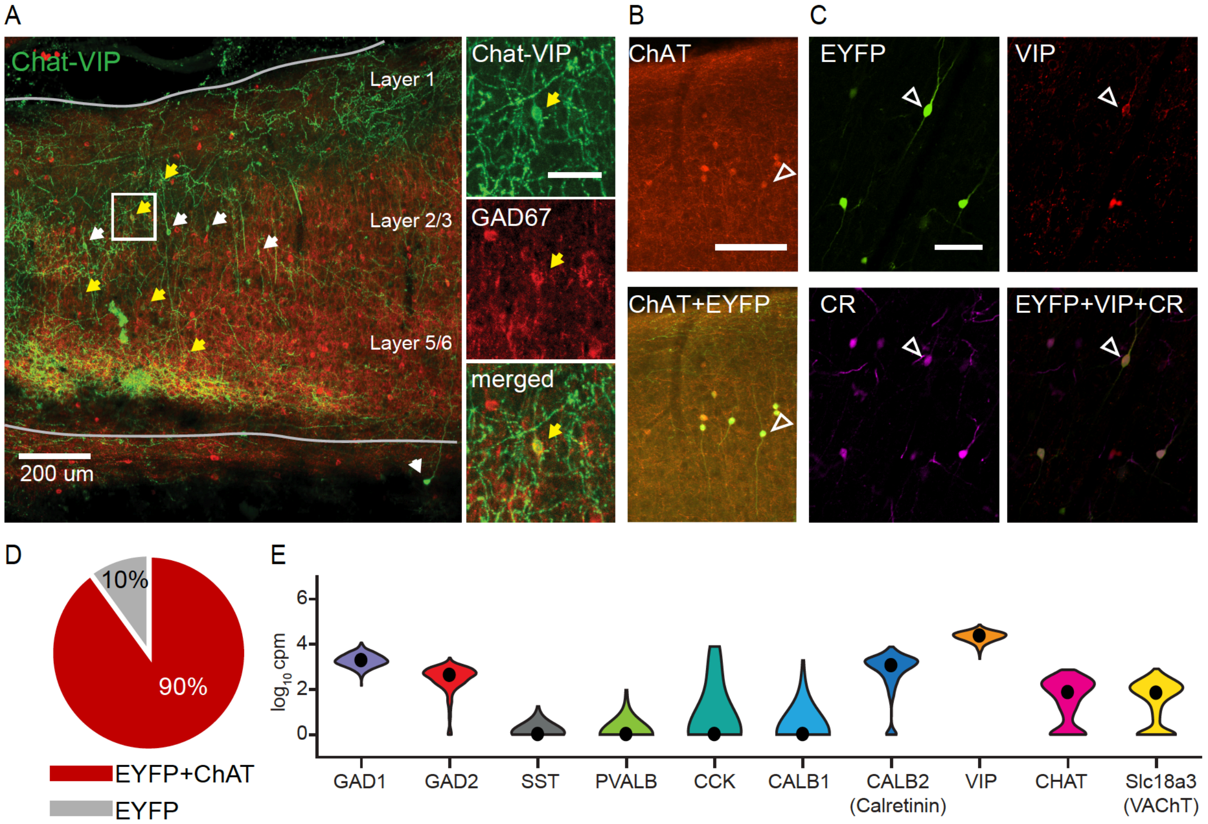

(A) EYFP-positive interneurons in mPFC of ChAT-cre rats stain positive with antibodies against choline-acetyl transferase ChAT and GABA-synthesizing enzyme GAD67, as reported by Bayraktar et al., 1997 and for mouse by Von Engelhardt et al., 2007. Yellow arrows point to neurons that are positive for both EYFP and GAD67 antibody labeling. White arrows: no detectable GAD67 antibody signal.

(B) EYFP-positive interneurons in mPFC of ChAT-cre rats are positively labeled by ChAT antibody.

(C) EYFP-positive interneurons in mPFC of ChAT-cre rats also stain positive with antibodies against VIP and CR, as reported by Eckenstein and Baughman, 1984, Bayraktar et al., 1997 and for mouse by Von Engelhardt et al., 2007. Not all EYFP cells were positive for VIP antibody staining. A similar finding was reported by Prönneke et al., 2015, who observed in Vip-ires-cre mice with TdTomato reporter, that VIP antibody staining did not 100% overlap with TdTomato expression (see Figure 2A and 2A' in Prönneke et al., 2015).

(D) In mPFC of ChAT-cre rats, 90% of EYFP positive cells were found positive for ChAT antibody staining (n=192, 6 animals).

(E) Single-cell mRNA expression of various cortical interneuron markers by ChAT-VIP interneurons (n=770 cells; cell types included: Vip Chat Htr1f, Vip Ptprt Pkp2 and Vip Rspo4 Rxfp1 Chat, see Tasic et al., 2018). Violin plots represent distribution of mRNA expression in counts per million (CPM) of introns + exons on a log scale. Data from the public Allen Institute for Brain Science cell types database (https://celltypes.brain-map.org).

Supplementary Figure 4: Basal Forebrain cells are not retrogradely labeled by virus injections in the mPFC.

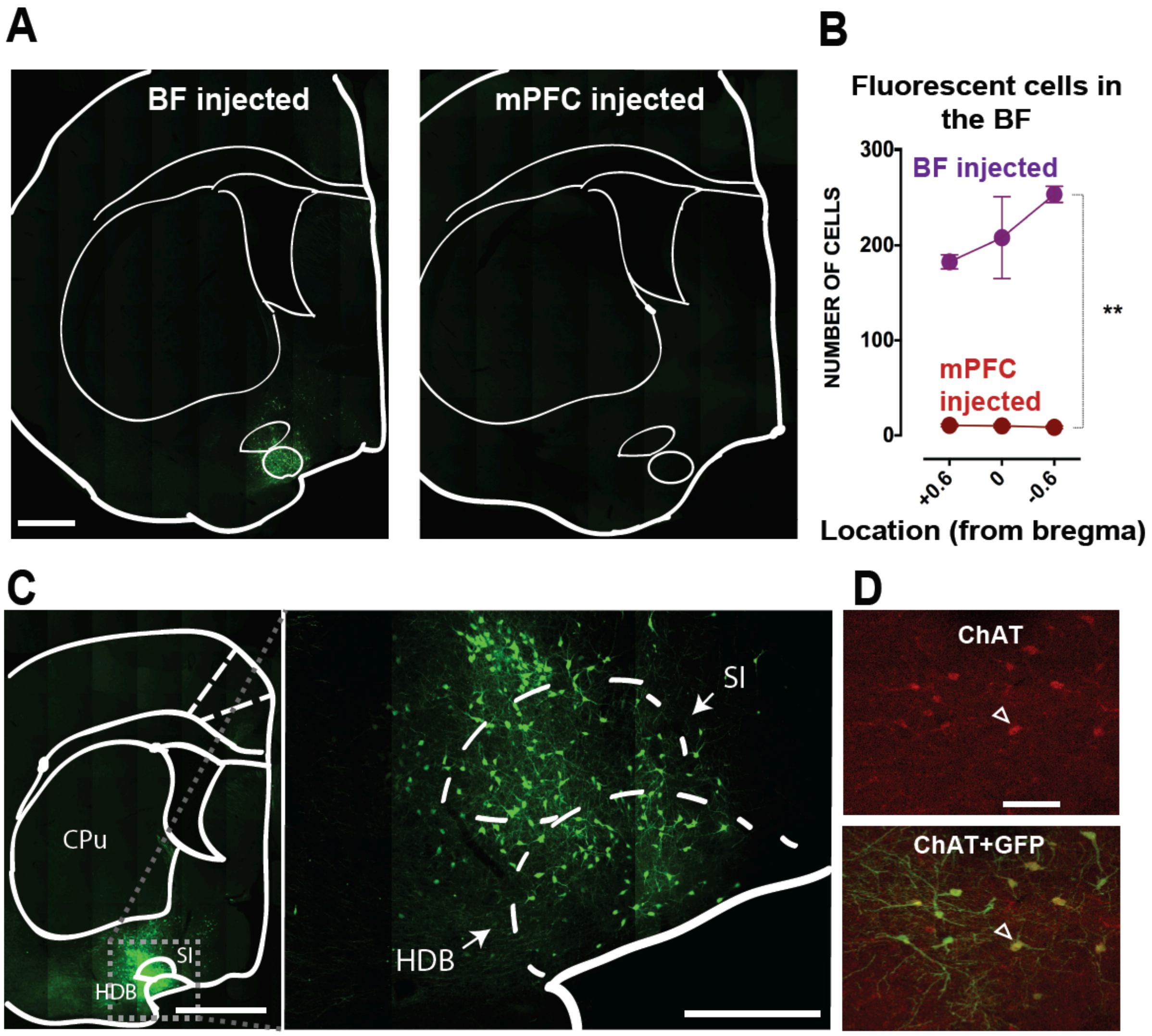

- **(A)** Basal forebrain (BF) expression of EYFP following AAV5 injection either in BF (left) or in the mPFC (right).
- **(B)** Number of fluorescent BF cells in BF injected vs mPFC injected ChAT::cre rats [effect of injection location: $F_{(1,2)}$= 518.1; p=0.001]. Summary plots show averages ± SEM. Scale bars: 1 mm (1A; 1C left panel); 500 µm (1C right panel); 200µm (E); 70µm (1D). Data are expressed as mean ± S.E.M, ** p<0.01.
- **(C)** AAV5 injections at the level of the HDB and SI (left panel and inset), labels ChAT-positive neurons and fibers in the mPFC. CPu: caudate putamen. DB: horizontal limb of diagonal band of Broca. SI: substantia innominate
- **(D)** EYFP-positive BF neurons of ChAT-cre rats are positively labeled by ChAT antibody.

Supplementary Figure 5: Co-transmission of GABA with ACh reduces spike probability of L1 interneurons.

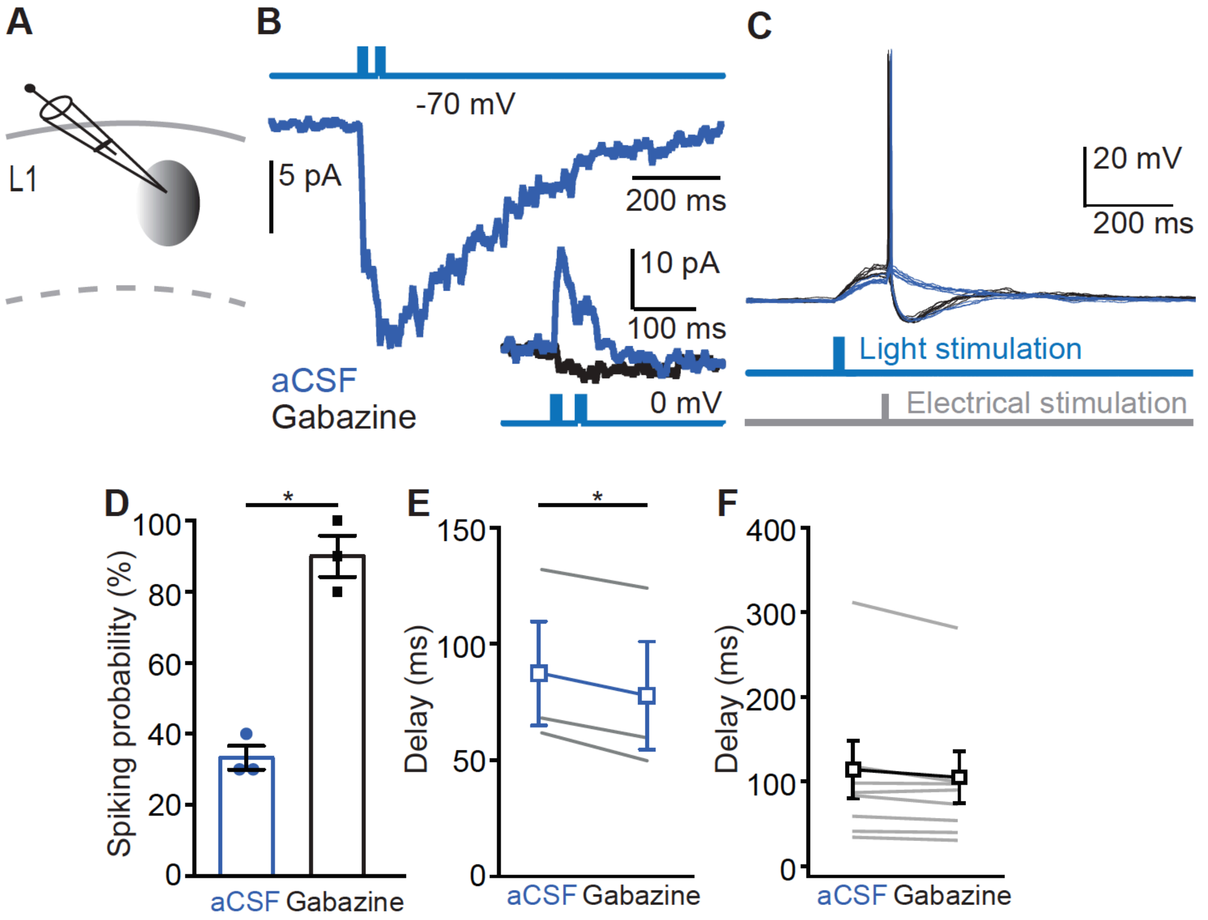

- **(A)** Schematic representation of the recording set up.
- **(B)** Light-evoked ChR2-mediated activation of ChAT-VIP cells generates an inward postsynaptic current response in a L1 interneuron recorded at -70 mV. Inset: same L1 interneuron recording showing a light evoked response at 0 mV in aCSF (blue trace) or Gabazine (black trace).
- **(C)** Example traces of a recording from a layer 1 interneuron. A short electrical stimulation (1ms) was combined with light evoked activation of ChAT-VIP interneurons. The electrical stimulation was adjusted in that way that the firing probability was ~30% in aCSF (blue trace). The spiking probability was measured again following wash in of Gabazine (black trace)
- **(D)** Summarizing the spiking probability in aCSF or Gabazine condition (aCSF 33.33±3.33%, Gabazine 90±5.77%, p=0.0234, paired *t*-test, two-tailed, t=6.425, df=2, n=3). Summary plots show averages ± SEM.
- **(E)** As in (**D**) summarizing the time to the first spike (aCSF 87.31 ±22.42ms, Gabazine 77.82 ±23.27ms, p=0.0179,paired *t*-test two-tailed, t=7370, df=3, n=3, mean ± S.E.M)
- **(F)** Summary graph of recordings from postsynaptic L1 interneurons in rat mPFC that only showed a nAChR current in response to blue light activation of ChAT-VIP neurons, similar to Fig 5A-F. Here, AP timing in response to ramp depolarizations were monitored without blue light stimulation and either in the absence (aCSF) or presence of gabazine. Gabazine did not advance spiking in these L1 neurons that did not show co-transmission of GABA.

Supplementary Figure 6: ChAT-VIP or BF projection inhibition during the 5 choice serial reaction time task (5CSRTT) does not affect omissions, impulsive or compulsive responses.

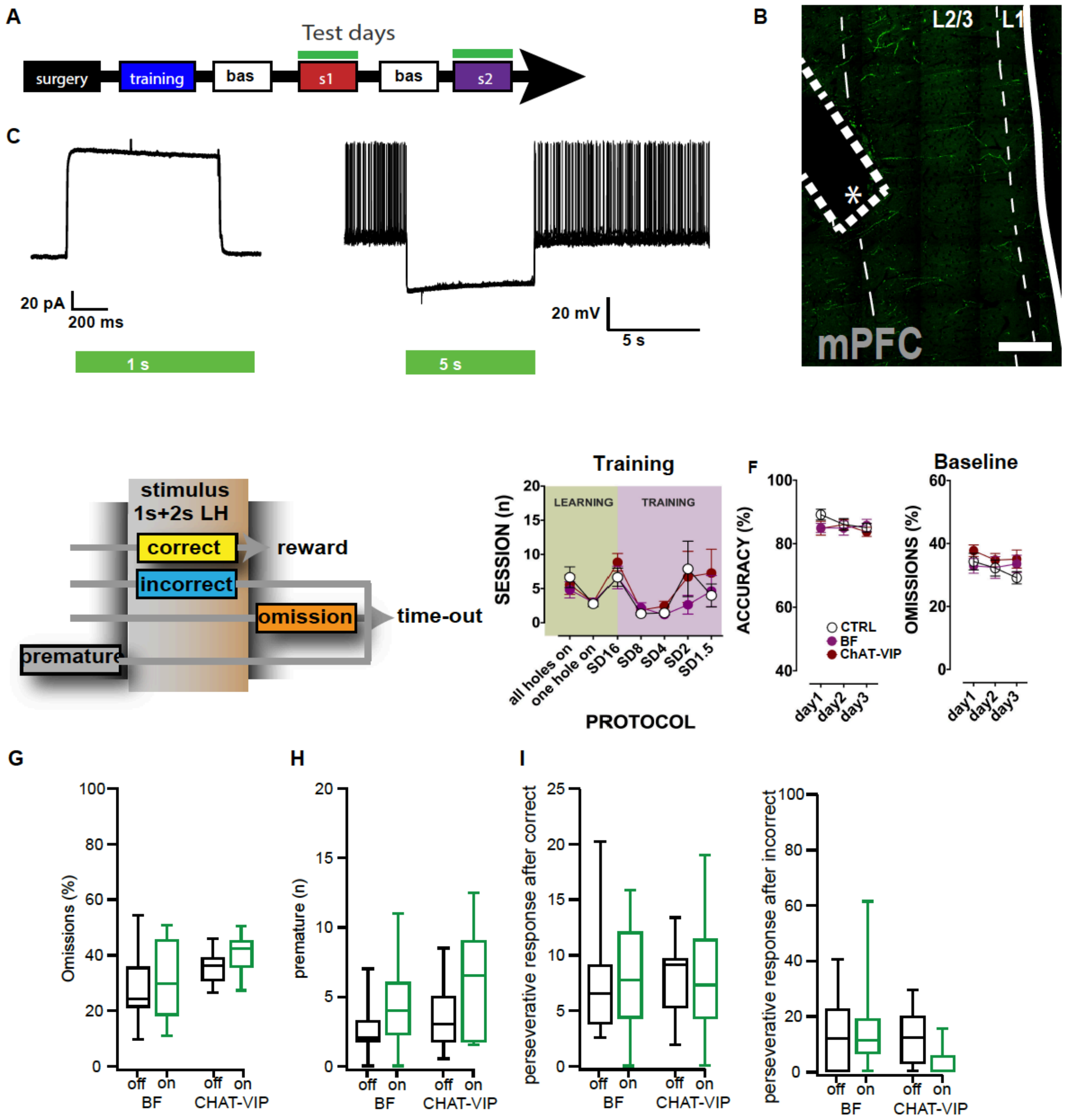

(A) Timeline of the behavioral experiments. Following surgery and recovery, rats were trained in the standard version of the 5CSRTT to stable baseline performance. Next, rats were tested for two sessions with random exposure to green light stimulation in the mPFC (S1 and S2). Between S1 and S2 rats underwent a session of the task without any laser light to test for potential carry-over effects of the light during the former session (BAS).

(B)) Optic fiber location for behavioral experiments. The asterisk indicates the optic fiber tip. In all animals, optic fibers were placed at the border of L2/3 and L5 of the mPFC. Scale bar: 200 µm.

(C) Whole-cell patch clamp experiments in acute brain slices of ChAT::cre rats injected with AAV5::DIO-ARCH3.0-EYFP used in behavioral experiments show prolonged

inhibition upon green light stimulation. Left: voltage-clamp recording of a ChAT-VIP interneuron shows a sustained inhibitory current upon green light exposure. Right: green light evoked hyperpolarization suppressed spiking activity.

(D) Response types during a trial in the 5CSRTT. Only correct hits are rewarded with food pellets while all the other responses received 5 second time-out period.

(E) Neither training duration across the different steps, nor the baseline (accuracy and omission, **F**) differ between the 3 groups (see inset in **F**).

(F) Neither training duration across the different steps, nor the baseline (accuracy and omission, **F**) differ between the 3 groups (EYFP control, mPFC injected labeling ChAT-VIP neurons, BF injected).

(G) Errors of omission did not differ when comparing laser-OFF and laser-ON trials, suggesting that both the BF and the ChAT-VIP interneurons play a negligible role in motivational aspects related to attentional performance. Box plot centre line: median; bounds of box: 25th to 75th percentile; whiskers: min-max data bound.

(H) Similar for premature responses

(I) and perseverative responses following correct trials (left) and perseverative responses following incorrect trials (right), which were not different in laser-OFF and laser-ON trials.
